# An Experimental Study of the Pull-In Voltage in RF MEMS Switches Fabricated by Au Electroplating and Standard Wet Release: Considering the Bridge Geometry

**DOI:** 10.3390/s25061877

**Published:** 2025-03-18

**Authors:** Loukas Michalas, George Stavrinidis, Katerina Tsagaraki, Antonis Stavrinidis, George Konstantinidis

**Affiliations:** 1 Institute of Electronic Structure and Laser, Foundation for Research and Technology–Hellas, 70013 Heraklion, Greece; gstav@physics.uoc.gr (G.S.); ktsag@physics.uoc.gr (K.T.); astav@physics.uoc.gr (A.S.); aek@physics.uoc.gr (G.K.); 2 Micro & Nano Technology Laboratory, Department of Electrical and Computer Engineering, Democritus University of Thrace, 67100 Xanthi, Greece; 3 Department of Physics, University of Crete, 70013 Heraklion, Greece

**Keywords:** RF MEMS, pull-in voltage, wet release, bridge geometry, high power, electroplating

## Abstract

Radio Frequency Micro Electro Mechanical Systems (RF MEMS) are devices showing exceptional potential to satisfy the demands of emerging RF electronic technologies, including those considered for high-power applications, such as for long distance communication systems. Operation in this regime requires an alternative way of thinking for these devices and, for example, a more accurate control of the pull-in voltage is of major importance due to the self-actuation effect. Therefore, the studies focusing on the features of the moving bridges are of great importance. This work presents the fabrication of a full family of RF MEMS switches suitable for high-power implementations having bridges deposited by Au electroplating and released using purely standard wet processes, as well as a carefully designed experimental study of their pull-in voltage. Depositing the bridge of the high-power RF MEMS by using only a single electroplating step makes the device fabrication easier, whilst the utilization of a purely wet release process is an asset. This method relies on low temperature processes, applicable simultaneously in bridges with various geometrical and perforation details without the need of any specialised infrastructure. The experimentally obtained results suggest that for this technology the bridge thickness is a critical factor for controlling the pull-in characteristics between devices fabricated in the same run. Moreover, it is revealed that for thicker bridges, geometry and hole perforation effects are more pronounced. This technology is therefore suitable for developing RF MEMS where the bridge thickness could be potentially utilized for enabling optimization engineering between devices that should be fabricated in the same run but need to satisfy diverse specifications during their operation.

## 1. Introduction

Radio Frequency Micro Electro Mechanical Systems (RF MEMS) appeared more than 20 years ago and since their early era showcased their remarkable capability to satisfy the diverse requirements of modern telecommunications systems. This ability is still well accepted by the RF community and RF MEMS are nowadays seriously considered for functionalities related to several envisioned applications either supported by the upper part of the microwave frequencies band or from systems requiring handling of high RF power levels, enabling next generations (e.g., 5G and beyond) and/or long distance (e.g., Radars and SatComs) communication systems [1,2,3,4,5].

Among the various types of RF MEMS switches, and without undervaluing others, the bridge type capacitive switches have the potential to address simultaneously the high power and the high frequency operation requirements. This is mainly owed to the features of the capacitive contact formed during their actuation [6]. The term actuation refers to the condition where the electrostatic force induced by an external stimulus is capable of driving the moving armature towards the bottom electrode. This is a fundamental phenomenon always worth being investigated in MEMS technologies and therefore was always a topic of interest for the RF MEMS scientific community including studies relying on electromechanical [7] or on analytical and/or numerical modeling and simulations [8] or by assessing experimental results [9], to name a few. Entering the high-power operation regime this interest should be further enhanced as the pull-in voltage is having a much more critical role.

Beginning with considering the low power implementations, the major research target related to the pull-in voltage (V_pull-in_) is typically focused on its minimization [10,11,12,13]. This demand stems mainly from the issue of dielectric charging that constitutes one of the most severe [14,15] and persisting [16] reliability constraints in RF MEMS technologies. Once the moving bridge will land atop the thin dielectric film in a capacitive contact, there will be an electrostatic field (E) with an intensity proportional to the actuation voltage (V_act_) and inverse proportional to the dielectric film thickness (d) as E = V_act_/d. The higher the field E, the more pronounced the dielectric charging effects and this has been demonstrated for diverse cases where different dielectric films such as SiO_2_ [17], Si_3_N_4_ [18,19] or micro/nano crystalline diamond are involved [20]. Therefore, considering the low power applications, a lower possible pull-in voltage is typically required.

Moving to the high-power operation regime the situation is completely inversed and an alternative way of thinking is required. In this case and for any specific power level the pull-in voltage should be higher than a minimum value to avoid the so-called self-actuation. This refers to the condition where the high-power RF signal itself is sufficient to pull-in the moving bridge without the need for any external stimulus [21,22]. This condition constitutes the most fundamental reliability issue for high-power implementations as it means that the specific switch that self-actuates as result of an RF signal passing through cannot effectively handle signals of this or higher power level. Therefore, the pull-in voltage should be higher than a specific value, defined by the details of the switch and the topology [6], for enabling applications at a specific power level. At the same time the pull-in voltage should not be extremely high because similarly to the low power operation regime, the filed intensity in the down state will accelerate the consequences of the dielectric charging issues, whilst it can also lead the dielectric film to breakdown. In addition, generating high electrostatic field has been experimentally demonstrated that can also lead to the onset of field emission currents even with the bridge in the up state such as in the case of fixed metallic MEMS-like structures [23,24], and in similar fixed MEMS-like structures that includes also the dielectric films [25], as well as in functional RF MEMS [26]. These currents may result in additional reliability issues and/or to lead to device failure [27]. Therefore, during both the low and particularly considering the high-power operation regime the control of the pull-in voltage is of major importance.

Despite the need for controlling the pull-in voltage with respect to the various RF applications, this is not always a straightforward task. For RF MEMS there are well defined analytical equations that include all the involved phenomena from the physics perspectives; however, it has turned out that the effects of the fabrication process on the mechanical properties of the suspending metallic and moving elements referred to as residual stress are quite complicated. The residual stress could be either naturally introduced during the deposition of the moving bridge or could be induced at the various process steps as materials with various expansion coefficients are coming in contact, whilst occasionally topography that exists in the lower parts of the devices should be followed and adapted by the upper deposited parts and especially from the movable ones. The release method typically performed either by plasma etching [28] or alternatively using a critical point dryer (CPD) [29] is also very critical and, overall, the effects of residual stress on the pull-in voltage are considered as very important by the community. This is evidenced by the numerous relevant studies focusing in diverse aspects as, for example, on assessing the residual stress in dry released beams through experimental results and analytical methods [30], or in similar structures through experimental results and Finite Element Modeling [31], or through simulations and calculations including the effects of holes perforation [32], or even on MEMS with cantilever beams [33,34], whilst the gained knowledge has been also summarized in relevant reviews [35]. Despite the numerous studies that undoubtably shined light on this multiparametric aspect, its nature is so complicated and in many perspectives case specific, and there are several features that are still requiring further investigation. This is particularly important in view of the high-power applications presently envisioned for the RF MEMS. To this direction, the dissemination of more experimental results stemming from carefully designed and focused studies will support deeper and better understanding of the related phenomena and should be considering as extremely useful towards the development of RF MEMS with controllable characteristics. This need is further enhanced when considering that with regards to systematic studies focusing on the effects of geometrical details of the moving bridges on the pull-in voltage, with emphasis on the process-induced residual stress, the available literature is still limited leaving room for more studies to come about. The latter also constitutes an extremely interesting topic as the bridge geometrical details apart from the mechanical response also defines the RF domain characteristics (through the capacitance, the inductance and the resistance of the device). Finally, another topic to be considered is that for most of the devices the release process is performed either by dry etching typically using oxygen plasma or by wet etching through a specialized equipment referred to as a critical point dryer (CPD).

Bearing these in mind, this work aims to present a carefully designed straightforward experimental study that reports both the bespoke fabrication of a family of RF MEMS capacitive switches, released through standard purely wet processes, and a targeted study of their pull-in voltage. The devices studied were fabricated based on a simple and commonly utilized topology considering X-band operation and by adopting the major features of the RF design presented in ref [36]. Further to these major features, the study included bridges of various thicknesses also having perforation from holes with different characteristic dimensions and distance between their centers referred to as pitch. The latter has been previously reported to affect both the residual stress and the elastic properties and their sizes and pitch could be very critical to this [6]. The RF MEMS tested were fabricated on the same wafer during the same process run and the only variation between these was the different thicknesses of the deposited bridges. The bridges were deposited in a single step by gold (Au) electroplating maintaining identical deposition conditions. The following release was performed also by using the same procedure based on standard (“purely”) wet processes (not using a critical point dryer—CPD), an approach not commonly adopted, which offers though several additional possibilities. Furthermore, their characterization was also carefully performed as presented in the corresponding section and the obtained results are discussed not only with respect to the pull-in voltage but bearing also in mind more generic device operation aspects.

## 2. Summarizing in Brief the Theoretical Background

In the case of a bridge type capacitive switch there are analytical expressions that define the pull-in voltage as a function of the various parameters involved. This is mostly expressed by some approximations resulting in the following equation [6]:

(1) $$V_{pi}=\sqrt{\frac{8kg_{0}^{3}}{27\varepsilon_{0}Ww}}$$

In the above equation *g*_0_ is the bridge height (i.e., the airgap spacing) when no bias is applied, *W* the width of the pull-down electrode (i.e., the signal line width of a coplanar waveguide- CPW), *w* the moving bridge width and *k* the spring constant of the fixed-fixed beam. Equation (1) is the most adopted one for expressing the pull-in voltage and provides a very good representation of how the various parameters are involved on the electrostatic actuation; however, it should be noted that in this form the existence of a thin dielectric film atop the bottom electrode as well as of the fringing fields are neglected. Nevertheless, these contributions are generally considered to be minor. Regarding the bridge details, meaning the material and the geometry, these are incorporated in the spring contact *k*. The latter is generally assumed to consist of two components as follows [6]:

(2) $$k=k_{1}+k_{2}$$

The first component (*k*_1_) is expressing the contribution of the stiffness to the mechanical response of the moving bridge whilst the second term (*k*_2_) is included to incorporate the contribution of what is referred to as residual stress. This is massively dependent on the various process steps applied during the device fabrication. The typical expression in the case where the attractive force is distributed above the area of the central waveguide contactor with *x* defining these limits [6] and at the same time the residual stress *σ* exists as a result of tensile stress only is:

(3) $$k=32Yw{\left(\frac{t}{l}\right)}^{3}\frac{1}{8{\left(\frac{x}{l}\right)}^{3}-20{\left(\frac{x}{l}\right)}^{2}+14\left(\frac{x}{l}\right)-1}+8\sigma (1-v)w\left(\frac{t}{l}\right)\frac{1}{3-2\left(\frac{x}{l}\right)}$$

With *Y* being the Young’s modulus, *v* the poison’s ratio and *t* and *l* the thickness and the length of the bridge, respectively.

As discussed, the above expression is extracted for a very specific set of conditions, but it is presenting the major and most interesting features for the spring constant. The latter could be also affected by the presence of perforation by holes existing in most of the cases in bridge type switches. The existence of holes introduces additional influence in the spring constant by modifying the Young’s modulus, the mass of the bridge whilst it has been reported to reduce the residual stress [6]. Moreover, with regards to the up-state capacitance and the electrostatic force, the effect of the holes is compensated by the fringing fields that are covering the corresponding area and therefore is considered as negligible if their diameter is less than 3–4*g*_0_. From this information it could be concluded that the analytical expressions (1)–(3) provide indeed a very nice representation of the physics underpinning the bridge electrostatic actuation phenomenon nevertheless cannot consider additional contributions (e.g., holes with diameters >4*g*_0_ or cases where residual stress is not purely tensile) typically exists.

## 3. Experimental

### 3.1. RF MEMS Fabrication

Considering the complicated electromechanical nature of the actuation mechanism in bridge type capacitive MEMS, presented in the previous section in brief, and in order to perform this experimental study focusing on the pull-in voltage, a set of devices having dissimilar characteristics were fabricated with this intent. These were designed based on the typical shunt configuration, in coplanar topology, presented in [36], with additional variations including bridge perforation with holes. All the fabrication steps were performed within an ISO-certified cleanroom where after each consequent process step a quality control measurement was applied to ensure that any fabrication-induced deviation from the design was maintained within very strict limits.

**Figure 1:**
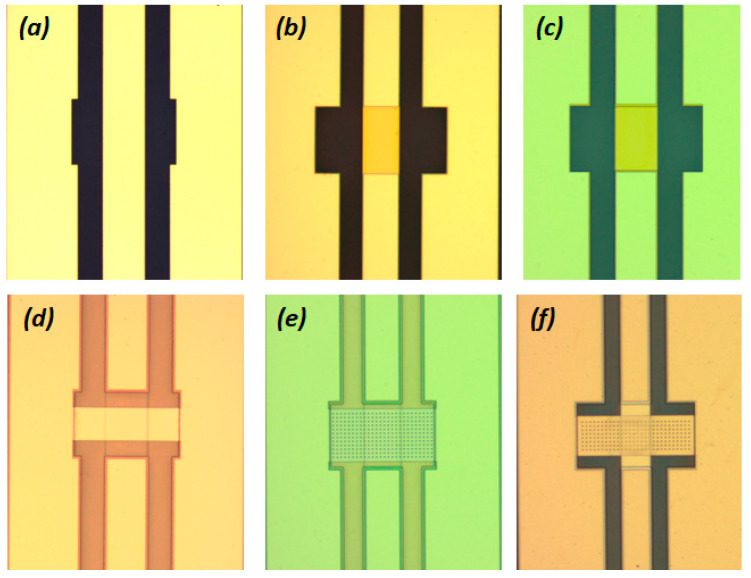
Optical microscope images showing the device at the various processes steps namely (**a**) deposition of the CPW lines, (**b**) patterning of the dielectric films, (**c**) deposition of the sacrificial layer, (**d**) deposition of the seed layer, (**e**) patterning the shape for the electroplating process step and (**f**) final devices after the bridge release. The devices presented above are having G/S/G (i) 65/110/65, (ii) 85/140/85 and 96/160/96 (in μm), bridge widths *w_1_* = 125 μm, *w_2_* = 150 μm, *w_3_* = 175 μm and *w_4_* = 200 μm, bridge length *l* = 450 μm and *l* = 500 μm, thicknesses *t* = 2.3 μm and *t* = 2.7 μm and holes with side dimension $a_{1}$ = 5 μm and $a_{2}$ = 10 μm.

The first fabrication step was the creation of the coplanar waveguide lines having the characteristic Ground/Signal/Ground (G/S/G) dimensions of (i) 65/110/65, (ii) 85/140/85 and 96/160/96 (in μm) as reported in [36] and that corresponds to the impedance Z_0_ = 50 Ω around 10 GHz. This was achieved by patterning the lines using optical lithography followed by e-beam evaporation of 300 nm Au (atop of 5 nm of Cr as adhesion layer) and lift-off process (Figure 1a). Then a 150 nm thick Silicon Nitride (SiN_x_) film was deposited (blanket deposition) in a Plasma Enhanced Chemical Vapor Deposition (PECVD) chamber. The following steps included the patterning through optical lithography and the selective removal of the dielectric film by Reactive Ion Etching (RIE), uncovering the Au lines for the following steps to be performed. Then the resist utilized as an etching mask was also removed (Figure 1b). These followed by the deposition of a positive photoresist as the sacrificial layer (Figure 1c), its patterning by suitable lithography (openings) through another patterned photoresist, the removal of the latter photoresist, the deposition of few nm of Cr/Au (5 nm/50 nm) seed layer by e-beam evaporation (Figure 1d) and the patterning through another photoresist of the shapes for the electroplating deposition to follow (Figure 1e). At this point, before the electroplating process the wafer was cut into pieces. Thus, the various devices to be tested should be identical up to this point including their airgap spacing that was approximately 2 μm according to the corresponding ISO quality control measurement performed for the corresponding photoresist thickness at different places on the wafer.

The moving bridges for the RF MEMS devices were deposited by utilizing a suitable commercially available solution of Au and by electroplating. Two different pieces having bridges with thicknesses of 2.3 μm and 2.7 μm were finally fabricated (Figure 1f). To allow a fair comparison in both cases, the deposition conditions were identical and suitable for yielding stress-free Au films. In both cases two variations in the bridge length (*l* = 450 μm and *l* = 500 μm) and four variations in the bridge width (*w_1_* = 125 μm, *w_2_* = 150 μm, *w_3_* = 175 μm and *w_4_* = 200 μm) were realized. The only difference between the two pieces was the deposition time duration that resulted finally in the bridges with the different thicknesses. Further to this it should be noted that in both pieces there were available devices having solid bridges (i.e., these are anticipated to obey the analytical expression) designed without holes, bridges designed to have holes of square shape with side size $a_{1}$ = 5 μm (thus diagonal $d_{1}=a_{1}\sqrt{2}<4g_{0})$ referred to as H1, and holes designed to have a square shape with a side size $a_{2}$ = 10 μm (thus both $a_{2}$ > 4*g*_0_ and the diagonal $d_{2}=a_{2}\sqrt{2}>4g_{0})$ referred to as H2. In both cases the distance between the holes was the same and consequently the distance between their centres referred to as the pitch was accordingly modified in the two cases. The mask-set included devices having different widths (*w*) and lengths (*l*) for the bridges of each variation mentioned.

The final fabrication step for the RF MEMSs was the removal of the sacrificial layer (followed the strip of the resist and the seed layer etching) and the bridge release. This was performed by a standard “purely” wet process (not with critical point dryer) using NMP in both cases. This method allowed for the realization of functional devices for all the available variations of the bridges existed on wafer. The implementation of a standard wet release method offers some additional capabilities that are also discussed in the following section.

### 3.2. RF MEMS Preliminary Characterization

**Figure 2:**
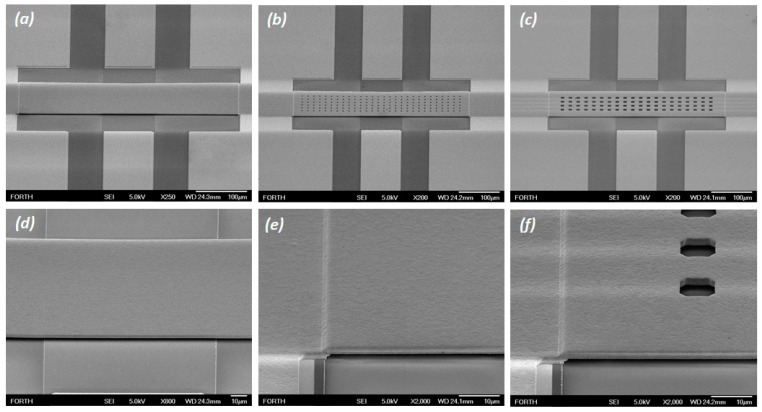
SEM micrographs showing the devices having (**a**) bridges without holes (FB), (**b**) bridges designed with holes side size $a_{1}$ = 5 μm (H1) (**c**) and $a_{2}$ = 10 μm (H2). The high-angle imaging (**d**–**f**) revealed that the bridges indeed remained suspended after the standard wet release process.

Once any RF MEMS fabrication is finalized two are the major features that should be primarily assessed. The first one is to confirm that the bridges remained suspended after the release process and if this indeed is the case then to evaluate if the suspended bridge is movable, i.e., it pulls-in and pulls-out successfully. To assess these aspects, some devices were initially characterized by Scanning Electron Microscopy (SEM). Figure 2 presents SEM micrographs of devices having bridges without holes (Figure 2a), with holes having sides with a size $a_{1}$ = 5 μm (Figure 2b) and also with a side size $a_{2}$ = 10 μm (Figure 2c). A close look under high-angle imaging confirms that the bridge remained suspended after the wet release process (Figure 2d) and a close look at the anchoring point (Figure 2e,f) shows the quality of the electroplated parts and the holes in detail. Overall, the SEM analysis confirmed the successful implementation of the fabrication process according to the design decided.

**Figure 3:**
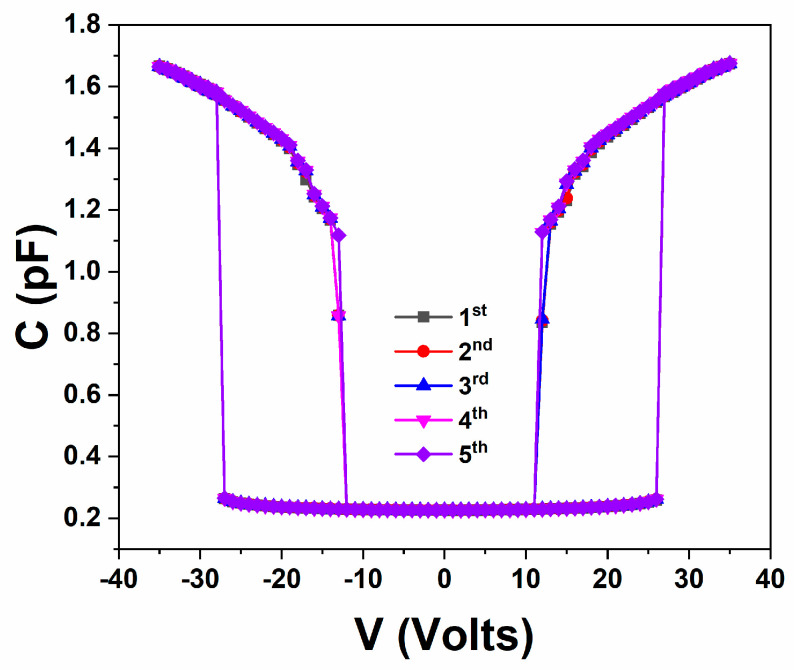
Typical capacitance-voltage (C-V) characteristics obtained (random day) from the fabricated RF MEMS switches. The presented one corresponds to a device fabricated on 65/110/65 (μm) lines, with *l* = 450 μm, *w* = 200 μm, *t* = 2.3 μm, having holes with side dimension $a_{2}$ = 10 μm.

Then selected devices from all the possible variations (though this study focused solely on RF MEMS fabricated on 65/110/65 (μm) CPW lines) were characterized electrically by performing on wafer capacitance-voltage (C-V) measurements with a small signal set at 1 MHz, using an LCR meter that provided also the DC bias voltage. All devices were functional and the Figure 3 presents a typical and representative response obtained on the RF MEMS capacitive switches. These devices clearly showed the ability to pull-in and pull-out effectively and to operate consistently. At this point it should be mentioned that the RF MEMSs were fabricated on a glass substate to avoid possible contribution from the Metal-Oxide-Semiconductor capacitor formed in the cases where Silicon substrate is utilized [37].

**Figure 4:**
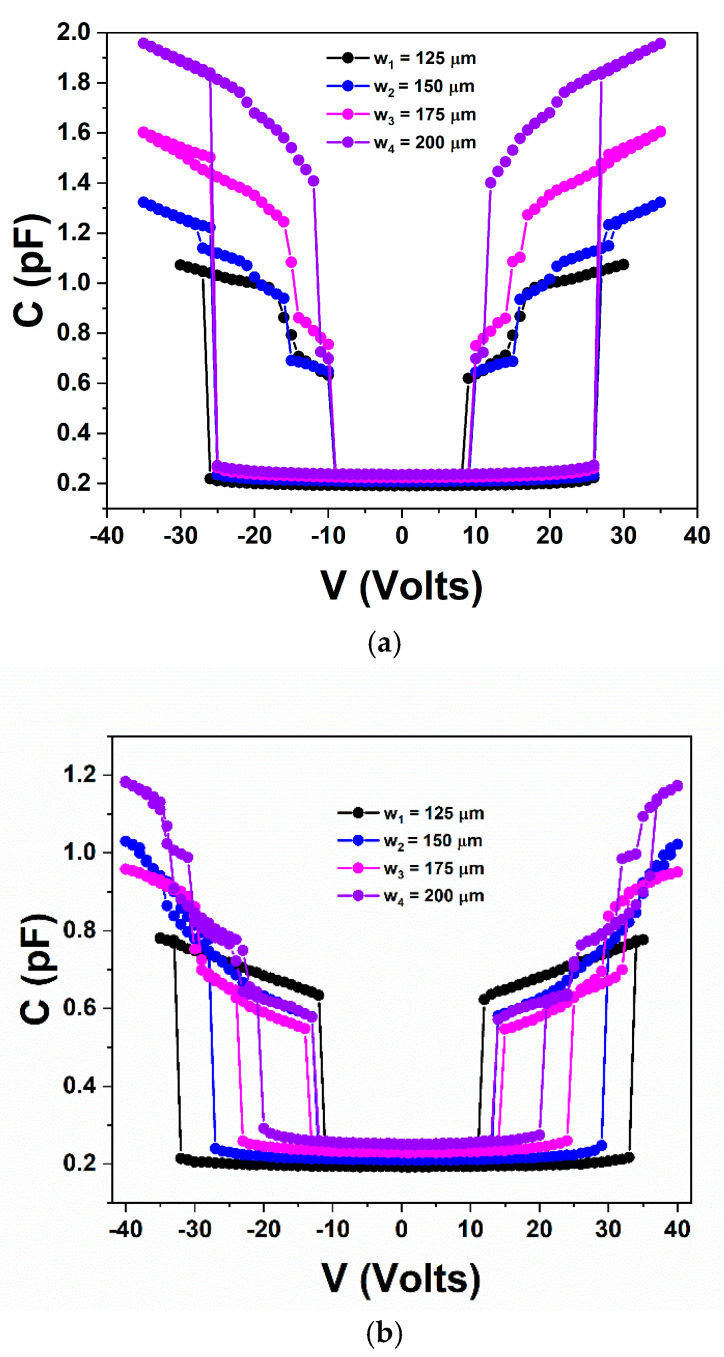
The preliminary capacitance-voltage (C-V) analysis performed multiple times (different days) on devices fabricated on 65/110/65 (μm) lines revealed two major trends. (**a**) Devices having bridges with a thickness *t* = 2.3 μm showing (practical) width-independent pull-in voltage or (**b**) devices having bridges with a thickness *t* = 2.7 μm and where the pull-in voltage exhibits a monotonic trend with the bridge width. In both cases the bridge length is *l* = 500 μm.

Finally, with regards to the pull-in voltage the preliminary assessment of the various devices fabricated on 65/110/65 (μm) lines revealed two major trends. The devices having bridges with a thickness of 2.3 μm showing bridge-width independent pull-in (identical or with slight random variations around one value) (Figure 4a) or devices with bridges of a thickness of 2.7 μm showing monotonic change of the pull-in voltage as a function of the bridge width (Figure 4b). Those two trends in the corresponding devices have been repeatedly obtained after multiple characterization campaigns performed on different days.

Overall, this preliminary assessment clearly demonstrated that the fabrication procedure resulted in fully functional devices. Thus, a more systematic study was performed in order to investigate the pull-in voltage characteristics. The details of this study as well as the results are presented in the following sections.

## 4. Results and Discussion

Assessing the pull-in voltage, although appears to be a quite straightforward and easy task, should be performed under a well-defined protocol to enable a fair comparison and to allow for extracting valid concluding remarks. This is because apart from the geometrical details, the pull-in voltage is also prone to other experimental parameters. Moreover, one should always bear in mind that this is defined by the properties of a micrometer scale moving metallic element, being suspended a few micrometers above the bottom electrode, thus even the same device may respond a bit different from time to time. Therefore, in this study emphasis is given mainly on the trends rather than on specific values, even though several details were taken into consideration as discussed in the following paragraph.

With regards to the aforementioned details, it should be always considered that the pull-in voltage is strongly affected by the dielectric charging [38]. Accumulation of charges in the dielectric films results in a shift of the capacitance-voltage characteristics, thus of the pull-in voltage. Therefore, in this study only the pristine (the first one) pull-in voltage was considered in the comparison of the various devices. The following pull-in voltages, after the first one, are strongly affected by the time duration that the bridge spent in the down state position, by the filed intensity at the down state position and by the time interval between two consequent actuations. Another parameter that has been reported to affect the mechanical response is the relative humidity [39]. In addition, the ambient temperature is also anticipated to play a role [40]. With these in mind and towards a fair comparison, before the systematic characterization, all the devices were placed overnight in the same vacuum desiccator. Then the devices were characterized during the same day, using the same setup, in the same laboratory, where the relative humidity and the ambient temperature were maintained practically constant. This was measured continuously with certified calibrated instrumentation.

The devices that were involved in this systematic characterization were all part of the same wafer and all based on transmission lines with the same characteristic dimensions (G/S/G) 65/110/65 (μm). Thus, all the fabrication steps up to the seed layer were common. All the steps underwent the ISO-quality control measurements that revealed practically no deviations from the targeted values. Consequently, the only expected variations are those in the bridge geometrical details. The protocol described above is expected to offer fair comparisons with regards to the trends appearing in the various devices involved in this study.

**Figure 5:**
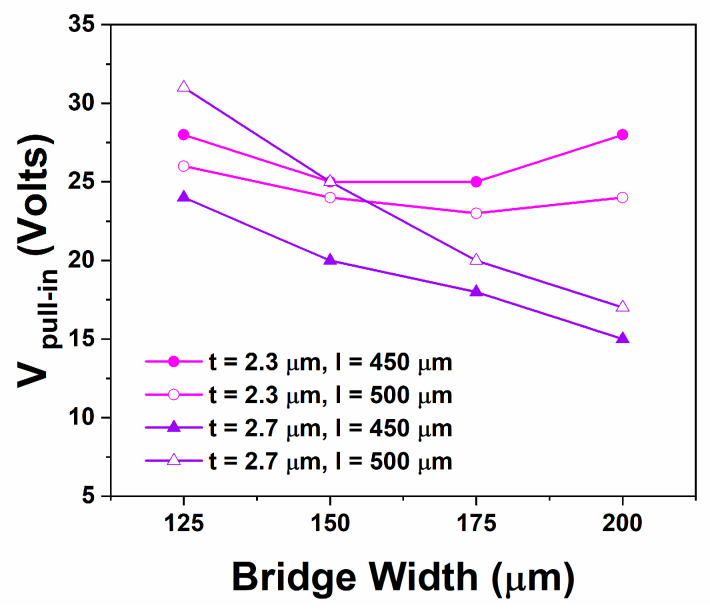
Experimentally obtained pull-in voltages for the devices with Full Bridges (FB) without holes.

Bearing these in mind, the initial discussion in this paragraph is focusing on the devices having bridges without any hole perforation referred to as “Full Bridge” (FB) in this work. These devices allow an assessment directly by the Equations (1)–(3). Figure 5 presents the experimental monitoring of the first pull-in, defined here for this comparison as the highest value of the applied voltage before the device first actuation, always performed with acquisitions towards positive biases. The results include for both bridge thicknesses mentioned above (2.3 μm and 2.7 μm), two variations in the bridge length (*l* = 450 μm and *l* = 500 μm) and four variations in the bridge width (*w_1_* = 125 μm, *w_2_* = 150 μm, *w_3_* = 175 μm and *w_4_* = 200 μm).

From Figure 5 it is clearly observed that bridges with different thicknesses result in different behaviour. Once more we wish to emphasize that our study is a straightforward experimental one that intents to focus more on the trends rather than to include detailed analytical calculations and exact values. Nevertheless, devices having bridges with a thickness of *t* = 2.3 μm appear to obey well the trends that are predicted by the theoretical Equations (1)–(3). The pull-in voltage is found to be practically width independent (without any clear trend) for both lengths (*l* = 450 μm and *l* = 500 μm) denoting that the anticipated width dependence predicted by both terms in Equation (3) (i.e., correspond to *k*_1_ and *k*_2_) is well followed in this case. With regards to the effect of bridge length, this comparison is more complicated as the term (*x/l*) is modified but also residual stress may vary between those two cases as the bridge should also follow the topography exist underneath. Thus, analysing and fully understanding the variations in the response between bridges having different lengths constitutes a greater challenge. Considering the MEMS devices having bridges with a thickness *t* = 2.7 μm, the response in these cases clearly deviates from the anticipated one according to the equations. There is a clear trend (observed consistently) for the pull-in voltage that decreases as the bridge width increases. Accepting that the stiffness of the bridge should increase due to the term (*t/l*)^3^ (i.e., correspond to *k_1_*), a possible reason for this trend is the existence of residual stress of the form *σ = σ (w)* that results in an overall reduction in the pull-in voltage and that deviates from the one existing in the previous case. Alternative (or complimentary) such responses could also be attributed (speculated) to deviations of the bridge shape from a purely flat one, e.g., on the formation of a curvature that results in a slight decrease of the effective gap (*g*_0_) and thus of the pull-in and that this effect scales with the bridge width (*w*). In similar speculated manner one may conclude that this non-flat shape along with the increased stiffness could be responsible for the lower pull-in voltage obtained in longer bridges (i.e., *l* = 500 μm over the *l* = 450 μm) in this case as well as to the absence of a clear capacitance trend in the down state like the one obtained in cases where the bridge thickness is 2.3 μm (Figure 4a).

**Figure 6:**
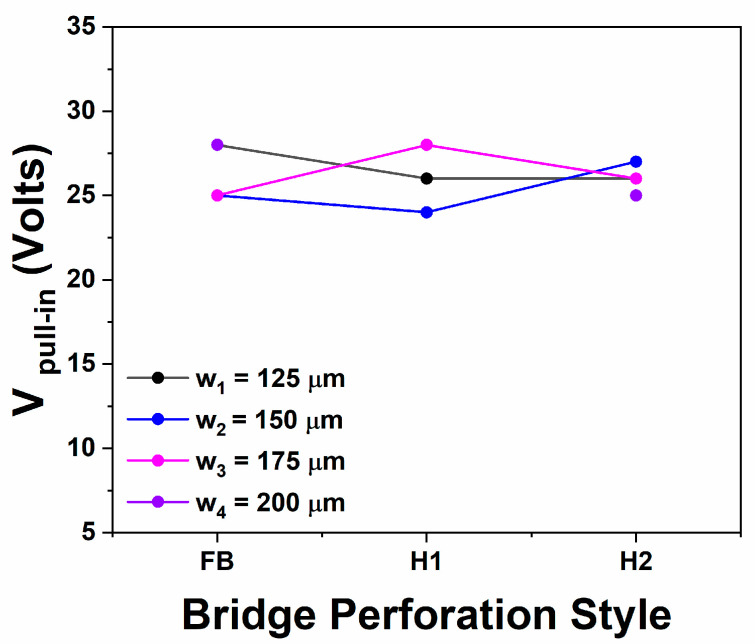
Experimentally obtained pull-in voltages for the devices having a bridge thickness of *t* = 2.3 μm and length *l* = 450 μm, including bridges without holes (FB), with small holes (H1) and with bigger holes (H2) as described in detail within the text.

**Figure 7:**
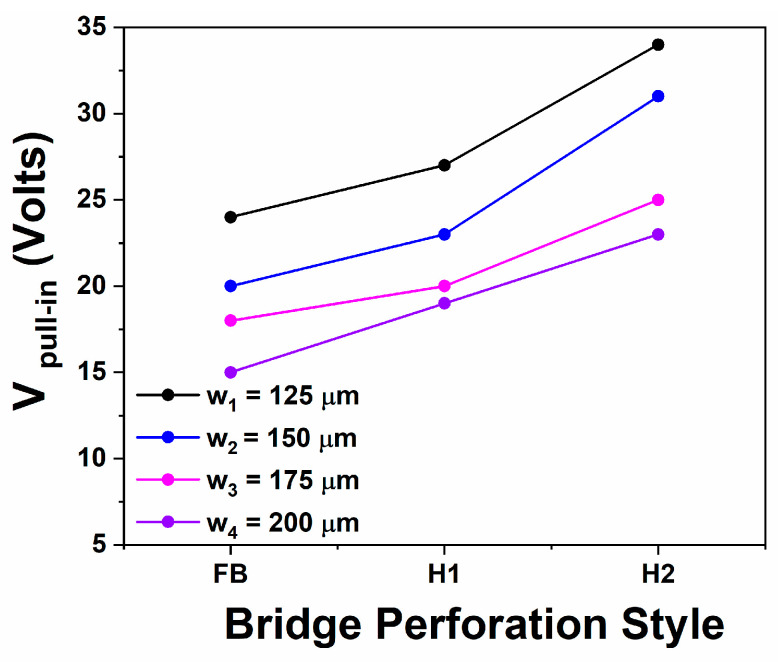
Experimentally obtained pull-in voltages for the devices having a bridge thickness of *t* = 2.7 μm and length *l* = 450 μm, including bridges without holes (FB), with small holes (H1) and with bigger holes (H2) as described in detail within the text.

Considering the cases where hole perforation exists on the bridges, similar to the above discussed trends are obtained, the pull-in voltage seems to remain practically unaffected (for the same *l* = 450 μm including all the various widths *w*) in Figure 6, corresponding to bridges having a thickness of *t* = 2.3 μm, whilst the effect of holes is clearly more pronounced in bridges presented in Figure 7 having a thickness of *t* = 2.7 μm (again for the same *l* = 450 μm including all the various widths *w*). Overall hole perforation is anticipated to offer some residual stress relief [6], while in the case of H2 (where both *a*_2_ > 4*g*_0_ and the diagonal $d_{2}=a_{2}\sqrt{2}>4g_{0})$ where the fringing fields probably do not fully cover the corresponding area, it should not be considered as unexpected an increase in the pull-in voltage, with regards to the FB case. The latter effect is clearly supported in cases where the thickness is *t* = 2.7 μm (H2 case) and it also may lead to the speculation that it compensates the residual stress relief in the case H2, where *t* = 2.3 μm, leading finally to a practically unaffected pull-in voltage. Considering case H1, this seems to create no effect in bridges of *t* = 2.3 μm, whilst a slight increase is obtained in cases where *t* = 2.7 μm. This fully confirms that residual stress effects in this case fully deviated from what is predicted by Equation (3) with potential origins those already discussed in the corresponding paragraph.

These results denote and confirm that the actuation features of RF MEMSs are heavily multiparametric. Under this understanding, performing predictions is not a straightforward task. Thus, this study generates the need for future, more thorough investigation with regards to the effects of bridge geometry (i.e., thickness, width, holes etc.) on the pull-in voltage as well as on the role of the overall device topology on pull-in voltage and residual stress effects [41]. Nevertheless, there are some clear experimental outcomes and few relevant perspectives that are worth being pointed out. Initially the development of RF MEMS switches through a standard wet release process (without CPD) of Au electroplated bridges is offering several advantages over the other typically utilized techniques (CPD and plasma etching). This include the fact that no specialized equipment is required, the fact that this is a low temperature process that does not have to comply with any potential area restrictions and that it enables to perform the moving bridge release in common process time and conditions independent of the bridge geometrical details (although thin bridges may collapse, thus may not be suitable for very low pull-in voltage applications). Finally, the absence of oxygen plasma during etching process results the minimum possible “contamination” of the Silicon Nitride dielectric. Further to these, it seems that the thickness of the bridge is a critical factor that allows designing according to the standard theory (i.e., *t* = 2.3 μm) or to deviate from this in other cases (e.g., *t* = 2.7 μm). The latter offers broad opportunities for engineering as it enables to facilitate devices with diverse characteristics in a common run and this could be extremely useful for RF MEMS related implementations. As an example of this, it can be mentioned that tuning the dimensions/perforation of the bridge may simultaneously allow for tunning the RF response (through the down-state inductance or capacitance in S-parameters) and the switch actuation requirements (through the pull-in voltage) related to the device reliability either through the power handling or to the dielectric charging related effects.

## 5. Conclusions

This paper reports the fabrication of a family of bridge type capacitive RF MEMS switches having bridges deposited by Au electroplating and released through standard wet processes (not with critical point dryer) as well as a carefully designed experimental study about the effect of the bridge geometrical details on the pull-in voltage. The utilization of standard wet release process enables the development of these devices without any requirement for specialized equipment, comply also with the low temperature manufacturability requirements and could be applicable to any type of bridge (except from very thin ones) including width and hole perforation in common process. The experimentally obtained results confirmed the successful device fabrication and their effective operation whilst with regards to the pull-in voltage, two major trends were revealed. For bridges having a thickness of 2.3 μm the results follow well the trends expected according to the commonly adopted theoretical equations. On the contrary, for thicker bridges of 2.7 μm, strong width and hole perforation influence on the pull-in voltage was obtained. These outcomes allow the design of applications targeting the high-power operation regime either according to the standard approaches (first case) or to consider engineering and optimizations between diverse switches having bridges (thicker, i.e., second case) deposited during the same run. In view of the above, the assessment of RF MEMS with bridges having various thicknesses and diverse topologies should constitute a following research theme.

## Data Availability

The data supporting this work are available at: https://elocus.lib.uoc.gr//dlib/1/4/d/metadata-dlib-1741867320-128073-4078.tkl (accessed on 10 March 2025).
